# A case report: Familial glucocorticoid deficiency associated with familial focal segmental glomerulosclerosis

**DOI:** 10.1186/1472-6823-12-32

**Published:** 2012-12-11

**Authors:** Nanik Ram, Ali Asghar, Najmul Islam

**Affiliations:** 1 Section of Endocrinology, Department of Medicine, Aga Khan University Hospital, Stadium Road, P.O. Box 3500, Karachi, 74800, Pakistan

**Keywords:** Familial glucocorticoid deficiency, Familial focal segmental glomerulosclerosis, ACTH resistance

## Abstract

**Background:**

Familial glucocorticoid deficiency (FGD) is a rare autosomal recessive disorder characterized by isolated glucocorticoid deficiency in the presence of normal plasma renin and aldosterone level. Focal segmental glomerulosclerosis (FSGS) is a form of glomerular disease associated with proteinuria and nephritic syndrome. This is the first case of familial glucocorticoid deficiency associated with familial focal segmental glomerulosclerosis.

**Case presentation:**

An eight month old boy presented with increased genital pigmentation. Initial investigation revealed that he was glucocorticoid deficient and was started on hydrocortisone and fludrocortisone with a diagnosis of primary adrenal insufficiency. Later fludrocortisone was withdrawn and he was diagnosed to have isolated glucocorticoid deficiency. He later developed focal segmental glomerulosclerosis for which he underwent renal transplantation at the age of five years. Now at the age of twelve years, this boy is doing well on hydrocortisone treatment. His two siblings and a first degree cousin also had isolated glucocorticoid deficiency. One of the above two siblings died due to renal failure secondary to focal segmental glomerulosclerosis.

**Conclusion:**

Patients with familial glucocorticoid deficiency should be carefully followed for development of features of nephrotic syndrome.

## Background

Familial glucocorticoid deficiency (FGD) is a rare autosomal recessive disorder characterized by isolated glucocorticoid deficiency in the presence of normal plasma renin and aldosterone level resulting from ACTH resistance [1]. Focal segmental glomerulosclerosis (FSGS) is a form of glomerular disease associated with proteinuria and nephritic syndrome [2]. Secondary FSGS is reported with endocrine disorders like Addison’s disease [3], cushing disease [4], acromegaly [5] and panhypopituarism [6].

We present here a rare case of a patient with FGD associated with familial FSGS. To our knowledge, there are no case reports of such patients.

## Case presentation

An eight month old boy brought by parents in clinic with complaint of increased genital pigmentation. Initial investigations showed low serum cortisol with an ACTH level 1250 pg/ml (6–48 pg/ml). He was started on steroid replacement of hydrocortisone and fludrocortisones with the diagnosis of primary adrenal insufficiency. He was doing well and repeated laboratory results revealed ACTH level of 7 pg/ml and 14.7 pg/ml on two occasions after steroid replacement. Later the diagnosis of primary adrenal insufficiency was re-visited based on laboratory results, when off fludrocortisone and hydrocortisone his serum cortisol decreased with highly elevated ACTH level but normal electrolytes and plasma rennin activity. His diagnosis was changed to isolated glucocorticoid deficiency.

At age of two and half years he developed generalized body swelling and facial puffiness. His baseline labs showed a serum creatinine of 0.5 mg/dl, serum sodium of 139 meq/L and a serum potassium of 4.0 meq/L. Endocrine workup revealed a ACTH level of 1179 pg/ml, cortisol of 6.15 μg/dl (2.8 to 23 μg/dL) and plasma renin activity of 3.35 ng/ml/hr (2.35 to 37 ng/mL/hour). The question was raised about compliance to medicine. Further labs were done for possible nephrotic syndrome based on his clinical presentation which showed a 24 hour urinary protein result of 3679 mg/24 hour with serum total protein of 3.6 g/dl, albumin of 0.9 gm/dl and a globulin level of 2.7 gm/dl. Steroid dose was increased but proteinuria did not respond and later immunosuppressive therapy was added.

Renal biopsy was undertaken and histopathology showed single core of renal tissue with up to 12 glommeruli. Of these four were totally sclerosed with reactive epithelial proliferation. Three showed segmental sclerosis, while five of them showed minor changes. There was no vasculopathy but tiny patches of mild tubular atrophy and interstitial sclerosis were seen. Immunoflorescense done on frozen tissue showed granular membrane and mesangial positivity of IgM, weak positivity of C3 andC1q in the same distribution as IgM while IgG and IgA were negative. In conclusion, light microscopy and IMF features were suggestive of focal segmental glomerulosclerosis.

Renal functions rapidly deteriorated and his serum creatinine reached a level of 4.9 mg/dl. He progressed to end stage renal disease (ESRD) within a year of diagnosis and was started on renal replacement therapy. He was on hemodialysis for one year and later received living related (uncle was donor) renal transplant at the age of five years. He is doing well after transplant and his ACTH level is 1.4pmol/L now on steroid replacement.

The patient’s elder brother had the same presentation of increased genital pigmentation. He was diagnosed to have primary adrenal insufficiency initially. Hydrocortisone and fludrocortisone was started which he took for around 7–8 years. His diagnosis was reviewed later in view of low serum cortisol, high ACTH level with normal plasma rennin activity and electrolytes when he was off medicines. The diagnosis was changed to isolated glucocorticoid deficiency. Currently he is on hydrocortisone replacement for the last 8 years without any problem with recent ACTH level within normal range. His kidney functions test and urine detail report are normal.

His younger brother also had same problem of isolated glucocorticoid deficiency, and he was on steroid replacement. He also later developed nephrotic syndrome with biopsy proven focal segmental glomerulosclerosis. He ultimately received renal replacement therapy but died at the age of four years, before he could undergo renal transplantation.

Patient’s mother also noticed same increased pigmentation on genitals of her nephew; his lab workup was consistent with isolated glucocorticoid deficiency and was started on steroid replacement. He is now 3 years of age with no evidence of nephrotic syndrome so far.

**Figure 1:**
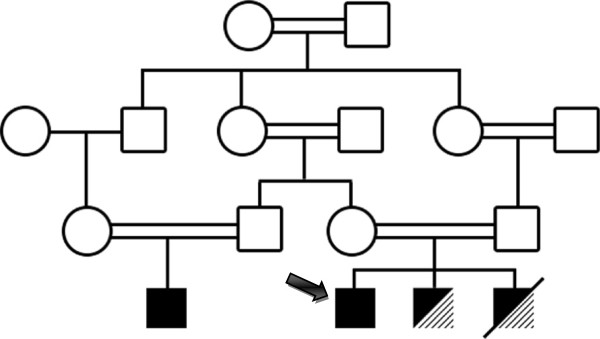
**Pedigree diagram.** Squares indicate males and circles females. White symbols show unaffected individuals; black symbols show individuals diagnosed with Isolated familial glucocorticoid defeciency FGD; diagonal stripes, individuals diagnosed with focal segmental glomerulosclerosis FSGS; and combined black and diagonal stripes, individuals diagnosed with FGD-FSGS. Filled arrow indicates proband. A double line indicates consanguine marriage. A diagonal black line marks deceased subject.

Patient’s family is not recalling any such type of illness history in their predecessors, although there is strong history of consanguineous marriages in the family (Figure 1).

In view of patient and siblings clinical presentation with low serum cortisol and high ACTH level with normal plasma renin activity, biopsy proven FSGS in both of them and consanguineous marriages, diagnosis of familial glucocorticoid deficiency associated with familial focal segmental glomerulosclerosis has been made.

## Discussion

FGD typically presents between the neonatal period and early childhood with hyper pigmentation, hypoglycemia, failure to thrive, recurrent infections and seizures due to hypoglycemia. All these symptoms and sign are related to either high ACTH or low plasma cortisol levels. There could be a family history of another affected member and/or consanguineous marriage [7,8]. Our patient, his siblings and cousin presented with hyper pigmentation, but none of the other classical presenting feature. The relatives come from a family with a history of cousin marriages.

This disorder is caused by mutation in one of four genes. ACTH receptor [melanocortin 2 receptor (***MC2R***)], its accessory protein [melanocortin 2 receptor accessory protein (***MRAP***)] or ***STAR***, all these are involved in ACTH signaling-steroidogenic pathway. Recently meimaridou et al. [9] found mutations in [nicotinamide nucleotide transhydrogenase (***NNT)*** an antioxidant defense gene responsible for FGD. ***NNT*** gene encodes an integral protein of the inner mitochondrial membrane which appears to be of primary importance for reactive oxygen species ROS detoxification in adrenocortical cells. There is impaired adrenal steroidogenesis due to this defective oxidative stress response. These genetic defects account for 40–45% of cases only [10]. In present case genetic testing has not been done due to non-availability of DNA analysis in Pakistan for these gene mutations. The mainstay of treatment is glucocorticoid replacement.

FSGS is a major cause of ESRD as it is responsible for about 20% of underlying pathology in children with ESRD. FSGS is usually sporadic in origin. Walker R et al. reported first autosomal dominant familial FSGS [11]. In a study by Conlon PJ et al. [12], they identified 60 families with familial FSGS with vertical mode of transmission that suggested a genetic link. They described FSGS as autosomal dominant when multiple generations were affected; autosomal recessive when single generation was affected. They observed more aggressive disease and earlier presentation in families with single generation involvement, as in our present case family.

Yamada S et al. [13] reported a case of FSGS with isolated ACTH deficiency and reversible hypothyroidism. In this case moderate proteinuria and thyroid function improved after steroid replacement. Arrizabalaga et al. [3] reported two cases of FSGS with Addison’s disease in which proteinuria were resolved after steroid and mineralocorticoid replacement. Contrary to above in our case proteinuria and renal functions in fact worsened after steroid therapy through an unknown mechanism.

There are reports of multiple family members being affected by FSGS in association with other hereditary disorders like Charcot-Marie-Tooth disease [14], Laurence Moon Biedl syndrome [15], Craniomandibular Dermatodysostosis [16].

In our case the reported association is less likely to be a coincidence, since two of three siblings have both conditions. To date there is no report of multiple family members being affected by FGD and FSGS. To know the link between familial FGD and familial FSGS genetic studies are needed.

## Conclusion

Neonates and children with the diagnosis of primary adrenal insufficiency in the presence of normal mineralocorticoid activity should be evaluated for isolated glucocorticoid deficiency. Family counseling of such patients is important for avoiding consanguineous marriages to decrease the chances of FGD, particularly in the case of autosomal recessive transmission. Because of the association of FGD with FSGS as shown by us, any patient with FGD should be carefully followed for development of features of nephrotic syndrome.

## Consent

Written informed consent was obtained from the patient’s father for publication of this case report. A copy of the written consent is available for review by the Series Editor of this journal.

## Abbreviations

FGD: Familial glucocorticoid deficiency; FSGS: Focal segmental glomerulosclerosis; ESRD: End stage renal disease; ACTH: Adrenocorticotrophic hormone.

## Competing interests

The authors declare that they have no competing interests.

## Authors’ contributions

NR led the conception and design, acquisition of data, review of literature, and drafted the manuscript. AA reviewed the manuscript. NI gave the concept of research paper, and critically reviewed the manuscript. All authors read and approved the manuscript.

## Authors’ information

NR is Fellow of the College of Physicians & Surgeons of Pakistan. He is Fellow in Endocrinology, Diabetes & Metabolism, Department of Medicine, Aga Khan University Hospital. He was involved in the medical management of the patient.

AA is member of the Royal Colleges of Physicians of the United Kingdom. He is Fellow in Endocrinology Diabetes & Metabolism, Department of Medicine, Aga Khan University Hospital. He was also involved in the medical management of the patient.

NI is Fellow of the Royal College of Physicians of London. He is Professor & Consultant Endocrinologist, Department of Medicine, Aga Khan University Hospital. He is also the Director Endocrinology, Diabetes and Metabolism Fellowship Program, Aga Khan University Hospital. He was patient’s primary physician & endocrinologist.

## Pre-publication history

The pre-publication history for this paper can be accessed here:

http://www.biomedcentral.com/1472-6823/12/32/prepub
